# Types of sensitization to aeroallergens: definitions, prevalences and impact on the diagnosis and treatment of allergic respiratory disease

**DOI:** 10.1186/2045-7022-4-16

**Published:** 2014-05-01

**Authors:** Michel Migueres, Ignacio Dávila, Franco Frati, Angel Azpeitia, Yasmine Jeanpetit, Michèle Lhéritier-Barrand, Cristoforo Incorvaia, Giorgio Ciprandi

**Affiliations:** 1 Service de Pneumologie et Allergologie, Clinique de L’Union, Saint-Jean, France; 2 Allergy Department, IBSAL, University Hospital of Salamanca, Salamanca, Spain; 3 Stallergenes Italia SRL, Milan, Italy; 4 Stallergenes Iberica SA, Barcelona, Spain; 5 Stallergenes SA, Antony, France; 6 Allergy/Pulmonary rehabilitation, ICP Hospital, Milan, Italy; 7 Department of Medicine, IRCCS-Azienda Ospedaliera Universitaria San Martino, Genoa, Italy

**Keywords:** Polysensitization, Paucisensitization, Co-sensitization, Co-recognition, Cross-reactivity, Cross-sensitization, Polyallergy, Monosensitization

## Abstract

The type of allergic sensitization is of central importance in the diagnosis and treatment of respiratory allergic diseases. At least 10% of the general population (and more than 50% of patients consulting for respiratory allergies) are polysensitized. Here, we review the recent literature on (i) the concepts of polysensitization, paucisensitization, co-sensitization, co-recognition, cross-reactivity, cross-sensitization, and polyallergy, (ii) the prevalence of polysensitization and (iii) the relationships between sensitization status, disease severity and treatment strategies. In molecular terms, clinical polysensitization can be divided into cross-sensitization (also known as cross-reactivity, in which the same IgE molecule binds to several allergens with common structural features) and co-sensitization (the simultaneous presence of different IgEs binding to allergens that may not necessarily have common structural features). There is a strong overall association between sensitization in skin prick tests and total IgE values but there is debate as to whether IgE thresholds are useful guides to the presence or absence of clinical symptoms in individual cases. Molecular information from component-resolved techniques appears to be of value for diagnosis and treatment decisions. Polysensitization develops over time and is a risk factor for respiratory allergy (being associated with disease severity) and therefore has clinical relevance for treatment decisions. The subterm polysensitization has been defined as polysensitization to between two and four allergens. Polyallergy is defined as clinically confirmed allergy to two or more allergens. Single-allergen grass pollen allergen immunotherapy (AIT) is safe and effective in polysensitized patients, whereas multi-allergen AIT requires more supporting evidence. Given that AIT may be more efficacious in moderate-to-severe disease than in mild disease, polysensitization could be an indication for this type of treatment. There is a need for flowcharts or decision trees for choosing the allergens for AIT in polysensitized patients and polyallergic patients.

## Background

Allergic respiratory diseases (including allergic rhinitis (AR) and allergic asthma (AA)) are major public health issues, with high prevalence and significant burden [1-5]. The management of respiratory allergy is based on allergen avoidance (when possible) [6], treatment with symptomatic drugs (such as antihistamines, inhaled, intranasal and systemic corticosteroids, bronchodilators and leukotriene receptor antagonists [1,7-9]) and allergen immunotherapy (AIT). The latter is a guidelines-supported therapy for moderate-to-severe AR and mild-to-moderate AA in patients in whom pharmacological treatment is ineffective, poorly tolerated or unwanted over the long term [1,2,8,9]. Patients with respiratory allergy may be sensitized and clinically allergic to one or more allergens. There are no guidelines how to modulate AIT as a function of the number or nature of the patient’s sensitizations. In the United States, allergists tend to use mixtures of extracts to treat for all sensitivities identified as individually important in skin prick tests (SPTs). In contrast, polysensitized patients in Europe are typically treated with one or a few allergens deemed to be most clinically relevant [10-12]. There is an ongoing debate as to whether single- or multiple-allergen formulations of AIT should be prescribed.

We reviewed the recent literature in order to (i) identify definitions of polysensitization, paucisensitization, co-sensitization, co-recognition, cross-reactivity (also known as cross-sensitization) and polyallergy, (ii) assess the prevalence of monosensitization and polysensitization in the general population and in patients consulting physicians for respiratory allergy, (iii) evaluate the relationship between sensitization status and the severity of allergic disease, and (iv) describe the key factors for diagnosis and treatment.

## Methods

MEDLINE, Embase and the Cochrane Library were searched from January 1999 up until May 2013 using logical combinations of the following terms (in English only): allergy; co-sensitization, polysensitization, co-recognition, cross-reactivity, polyallergy, epitope, multiple-allergen, multi-allergen, single-allergen, mono-allergen. Only English-language publications were selected for review.

## Results

### Defining polysensitization, paucisensitization, co-sensitization, co-recognition, cross-reactivity, cross-sensitization, and polyallergy

Before sensitization to one or more allergens can be discussed, it is essential to consider the definition of “allergen” itself. In a glossary issued by the European Academy of Allergy and Clinical Immunology’s Immunotherapy Task Force, Alvarez-Cuesta *et al*. defined an allergen as “a protein or glycoprotein capable of binding immunoglobulin E (IgE)” [13]. A biochemist would consider that an allergen is a protein (such as Der p 1 or Der p 2, the major allergens from *Dermatophagoides pteronyssinus*) with a defined amino acid sequence and three-dimensional structure. Even then, the Der p 1 produced by *D. pteronyssinus* is composed of several variant proteins with slight differences in the amino- acid sequence or with other small (post-translational) modifications [14]. Ultimately, one must consider antigenic determinants or epitopes – unique, localized regions on the solvent-accessible surface of an antigen that are capable of eliciting an immune response (and subsequently binding IgE). This distinction explains why cross-sensitization and co-sensitization (as discussed below) have different molecular bases and frequencies of occurrence. In 2004, Ferreira *et al*. proposed the definitions of “cross-reactivity”, “co-sensitization” and “co-recognition” [15]. They noted that allergen cross-reactivity is essentially related to similar protein three-dimensional structures and occurs when IgE antibodies are originally raised against one allergen and then bind to a similar protein in another allergen; this is also referred to as “cross-sensitization” in the literature. In general, more than 70% amino acid sequence identity is required for cross-reactivity. The authors listed 28 major groups of cross-reactive proteins from various sources, and stated that the interaction of serum specific IgEs (ssIgEs) with a cross-reactive, homologous protein may or may not trigger allergic reactions, depending on host factors, the allergen in question and the nature of the exposure. In the case of pollen allergy, if the patient does not have clear clinical symptoms related to a specific pollen period, it is impossible to know which of the two or more cross-reactive proteins the patient was first exposed to. Hence, Ferreira *et al*. suggested that the term “co-recognition” could define the great majority of IgE reactivity reactions in which the initial sensitizer is not known. They used the term “co-sensitization” to describe multiple, unrelated sensitizations to several structurally unrelated allergen groups. The structural basis of cross-reactivity was recently confirmed by Pfiffner *et al*. [16] in a micro-array study; 3,143 serum samples were tested to see whether they contained IgE that bound to any of 103 highly purified natural or recombinant allergens immobilized on the array. The researchers confirmed the previous cross-reactivity data from conventional IgE assays and reported predictions of cross-reactivity based on an iterative, motif detection algorithm. They hypothesized that cross-reaction (i.e. a single type of IgE binding to two or more related motifs) was more common than co-sensitization (i.e. separate IgEs each binding to different motifs). They also described a “hierarchy” of allergens within motif groups and speculated that this might influence the choice of AIT formulation. These observations also raised the issue of whether AIT should employ the most frequently cross-reacting allergen or an allergen that most stringently cross-reacted with other allergens.

The term “co-recognition” (for IgE reactivity reactions in which the initial sensitizer is not known) will typically apply to the particular case of panallergens [17]. These are groups of evolutionarily conserved proteins found in many different plant genera and that present a very high degree of molecular homology. Essentially, each member of a panallergen family is co-recognized with each other member. The best-known panallergens are the profilin proteins, the polcalcin calcium transport proteins, the lipid transfer proteins and tropomyosin [17]. Although these panallergens are not highly abundant, the high degree of homology can confound diagnoses. For example, the ssIgE against the profilin Phl p 12 in subjects with grass pollen allergy can also bind to Hev b 8 from latex extract – though this is not associated with clinically relevant latex allergy [18]. Non-protein molecules can also induce IgE-driven cross-reactivity; cross-reactive carbohydrate determinants are glycoprotein-borne asparagine-linked oligosaccharides that can variously be found in insect venoms, plant pollens, mites, crustaceans and vegetables [19].

Strictly, “polysensitization” means “more than one sensitization”, i.e. anything other than monosensitization. However, we note that de Jong *et al*. proposed to use the term “paucisensitization” to describe 2 to 4 sensitizations and “polysensitization” to describe 5 or more sensitizations [20]. Although this distinction may be relevant for certain clinical studies, only a small proportion of patients consulting allergists will be sensitized to 5 or more allergens, and we consider that “monosensitization” and “polysensitization” are sufficient for clinical use.

In summary, polysensitization can be divided into (i) cross-reactivity/cross-sensitization (the same IgE binds to several different allergens with common structural features) and (ii) co-sensitization (the simultaneous presence of different IgEs that bind to allergens that may not necessarily have common structural features). These relationships will be of importance when considering component-based diagnostics (CRD). Our definitions of polysensitization, paucisensitization, co-sensitization, co-recognition, cross-reactivity/cross-sensitization and polyallergy are given in Table 1.

**Table 1:** Summary of definitions

**Term**	**Definition**
Polysensitization	Sensitization (as confirmed by SPTs or ssIgE assays) to two or more allergens
Paucisensitization	Polysensitization (as confirmed by specific SPTs or ssIgE assays) to between two and four allergens.
Co-sensitization,	IgE reactivity reactions in which multiple, unrelated sensitizations arise against structurally unrelated allergen groups.
Cross-sensitization/cross-reactivity,	IgE reactivity reactions in which IgE antibodies are originally raised against one allergen and then bind to a similar protein in another allergen
Co-recognition	A subset of cross-sensitization/cross-reactivity reactions in which the initial sensitizer is not known.
Polyallergy	Clinically confirmed allergy (i.e. specific sensitization in SPTs or ssIgE assays and a causal relationship between symptoms and exposure to a specific allergen) to two or more allergens

### The prevalence of monosensitization and polysensitization in the general population and in patients consulting for respiratory allergies

There is a large body of evidence to show that polysensitization (as judged by SPTs or ssIgE assays) is common in the general population worldwide. In the first European Community Respiratory Health Survey (ECRHS) of 11,355 subjects, between 57% and 67.8% of the study population (depending on the country in question) were not sensitized to any of the test allergens, 16.2% to 19.6% were monosensitized to one test allergen and 12.8% to 25.3% were sensitized to two or more test allergens [21]. Similar results were obtained in the USA with SPT data from a sample (n = 10,863) of the general population (aged from 6 to 59 years) studied in the third National Health and Nutrition Examination Survey: 45.7% were not sensitized to any of the test allergens, 15.5% were monosensitized and 38.8% were polysensitized [22]. A number of studies (Table 2) have revealed even higher prevalences of polysensitization in patients consulting physicians for respiratory and food allergies [22-26]. In the study by Ciprandi *et al*., clinical symptoms were more severe in polysensitized patients than in monosensitized patients [26]. In addition, in a study of SPT results in 3225 AR patients from Spain and Portugal, the mean ± SD number of positive tests per patient was 6.5 ± 2 [27]. In a US study of 1338 patients with objectively diagnosed mild-to-moderate asthma, 81% reacted to three or more allergens [28].

**Table 2:** Studies reporting the prevalence of polysensitization

**Author (reference)**	**Population**	**% of polysensitized patients**
de Jong *et al*. [22]	9044 children referred to a central lab for sensitization testing	27.5%
Migueres *et. al*. [23]	2714 AR patients	73.5%
Didier *et al*. [24]	4227 AR patients	62.0%
Navarro *et al*. [25]	4991 AR patients	31.0%
Ciprandi *et al*. [26]	2415 AR patients	74.3%

These data make clear that polysensitization is highly prevalent in allergic patients, although the rates depend on the population and the region. Patients tend to gain sensitizations over time. Hence, it is not possible to directly compare rates obtained in different studies. Monosensitized subjects may correspond to children who will become polysensitized later in life or, more rarely, adults who will never develop additional allergen sensitizations. In 1999, Silvestri *et al*. published a study of changes in allergic sensitization in 165 monosensitized children with asthma (aged from 18 months to 8 years at enrolment) [29]. All the subjects were tested twice with SPTs for HDM, pollens, animal dander and moulds at time intervals ranging from 2 to 10 years. It was found that 43.6% of these initially monosensitized children had become polysensitized at their second visit (47.9% of those aged less than 5 years and 37.3% of the older children). The changes over time were statistically significant and varied from one allergen to another: for children initially monosensitized to HDMs, 45.4% became polysensitized, whereas the equivalent figure for children initially monosensitized to pollens was 32.1%. Hatzler *et al*. studied the time course of sensitization to *Phleum pratense* allergens in the German Multicentre Allergy Study by collecting ssIgE at the ages of 1, 2, 3, 5, 6, 7, 10, and 13 [30]. The results showed that IgE sensitization at the age of 3 years predicted AR by the age of 12 years and that a chronological order for the development of sensitization to Phl allergens (with Phl p 1 as the initiator in more than 75% of cases, followed by Phl p 4 and Phl p 5) was apparent. An overall assessment of epidemiological and clinical trial data led a recent review by Calderon *et al*. [10] to conclude that 51–81% of patients with allergies are polysensitized.

### The relationship between sensitization and clinical allergy

Sensitization to one or more allergens is highly prevalent but does not indicate a clinically relevant allergy. When establishing the clinical relevance of a sensitization, it is essential to compare the results of SPTs and/or ssIgE assays with strong circumstantial evidence (such the patient’s reporting of the effect of allergen exposure) or a direct allergen challenge (nasal, conjunctival or bronchial) [9]. In a review article published in 2006, the GA2LEN working group examined factors responsible for differences between IgE-sensitized asymptomatic subjects and IgE-sensitized allergic patients [31]. The authors emphasized that among individuals with ssIgE, a consistent number do not have clinical allergic disease. For example, 43% of the subjects with ssIgE to inhalant allergens in the Dutch part of the ECRHS did not present respiratory symptoms. There are many reasons for this difference, including a family history of atopy, levels of total serum IgE and ssIgE or IgG, the epitope specificity of ssIgEs, monosensitization vs. polysensitization, unidentified serum factors, the Treg/Th1/Th2 balance and IgE receptor polymorphisms and activation [31]. However, none of these are “all or nothing” factors. Burbach *et al*. examined data from 3034 patients in a multicentre, open, pan-European GA2LEN study [32] and distinguished between the standardized sensitization rate (SSR, i.e. all positive SPTs) and the clinically relevant sensitization rate (CCR, in which patients were asked whether they had symptoms in response to exposure to the allergen). The SSR to a particular allergen extract varied markedly from one country to another. The CCR ranged from 50% and 95%, depending on the allergen and country in question. In terms of the relationship between polysensitization and disease, the risk of AR increased dramatically with the number of sensitizations and was greatest for children with 5 or 6 sensitizations (adjusted odds ratio (OR): 12.73) and adults with 7 or more sensitizations (adjusted OR: 21.89. Having 7 or more sensitizations was also associated with a high risk of AA (adjusted OR: 6.12 in children and 5.65 in adults) and food allergy (adjusted OR: 6.93 in children and 2.61 in adults).

The “POLISMAIL” series of studies (for a review, see Ciprandi *et al*. [33]) has also characterized the sensitization status of patients consulting allergists. The first POLISMAIL study evaluated the clinical characteristics of 418 patients with AR. Only 10% of the patients were monosensitized and the mean number of sensitizations at the start of the study was 2.6 [34]. In another study, the researchers studied serum IgE, IgG, IgG_4_ and IgA levels for HDM, birch, grasses and Parietaria allergens in 80 polysensitized patients with AR. The immunoglobulin pattern differed slightly for each allergen, and a positive correlation between IgE levels and disease severity in the study population was found [35]. According to de Jong et al, the strong association between sensitization status and total IgE values and the striking co-sensitization between biologically unrelated allergens suggest that polysensitization is the expression of a distinct clinical, more severe, atopic phenotype, rather than of biological cross-reactivity to similar allergens [22]. Fasce *et al*. studied 98 infants (aged under 12 months at baseline) with onset of wheezing and concluded that respiratory allergy always starts with monosensitization [36]. All children underwent SPTs at baseline and after 2 and 5 years. At baseline, none of the infants was polysensitized, 20% were monosensitized and 80% were non-sensitized; 5 years later, more than 60% of the infants were polysensitized or monosensitized (mainly to HDMs). There was not always a direct relationship between an increase in the number of sensitizations and the prevalence of wheezing; some children grew out of their wheezing but continued to develop sensitizations.

### Diagnostic approaches in polysensitized and polyallergic subjects

When assessing a polysensitized patient, one of the physician’s main tasks is to establish whether the individual is truly polyallergic. The diagnosis of distinct clinical allergies can be facilitated by considering (i) the period during which the symptoms appear (e.g. spring for tree pollens, early summer for grass pollens and late summer for weed pollens), (ii) the potential presence of oral allergy syndrome (e.g. apple oral allergy syndrome is strongly associated with the presence of an existing birch pollen allergy), (iii) very large differences in the relative titres of the various ssIgEs and (iv) the outcome of nasal or conjunctival challenge tests, if reliably available.

When performed correctly, SPT is an important tool for the diagnosis of allergy and is highly specific and sensitive for the diagnosis of sensitization to inhalant allergens. A positive SPT does not, however, always correlate with clinical symptoms and the clinical relevance of a positive SPT must always be established before treatment is initiated [32]. Bousquet *et al*. have stated that despite the overall correlation between the degree of IgE sensitization and the risk of allergic symptoms, IgE thresholds are far from being absolute guides to the presence or absence of clinical allergy; in addition, some sensitizations are best detected with SPTs and others with ssIgE assays [37].

It is important to consider sensitization to panallergens when reviewing SPT vs. IgE assay results. Orovitg *et al*. compared the results of SPTs for grass, tree and weed pollens with IgE assays (on the ADVIA-Centaur platform) for specific allergens (including purified panallergens) in 179 polysensitized, pollen-allergic patients in Spain [38]. Both SPTs and ssIgE assays indicated that sensitizations to olive and grass pollens were frequent. The major grass allergen Phl p 5 was identified as a clear risk factor for panallergen profilin sensitization (OR: 8.3). The authors concluded that sensitization to panallergens could be a confounding factor in the diagnosis of polysensitized pollen-allergic patients. In a study of CRD, Barber *et al*. found that patients who were simultaneously sensitized to polcalcins and profilins had twice as many sensitizations to major allergens as patients sensitized to only one group or the other [39].

It is apparent that sensitizations are better dissected with ssIgE assays than with SPTs. In a study of 494 polysensitized children in whom SPTs had failed to reveal the causal allergen, Ciprandi *et al*. noted significant differences between the various ssIgE levels tested and thus suggested that IgE assays are a more appropriate measurement in this context [40]. Technical progress and the lack of absolute concordance between SPTs and ssIgE assays have stimulated the development of CRD as a novel *in vitro* method for assaying allergen sensitization [41,42]. This method quantifies ssIgEs that bind to single allergenic protein components (purified from natural sources or obtained by recombinant techniques) or even peptide fragments of allergenic proteins, rather than whole extracts of native allergens. There is evidence to suggest that (i) CRD can identify clinically significant ssIgE, (ii) some sensitization patterns are associated with particular prognostic outcomes and (iii) CRD can help the physician to choose the optimal allergen (s) for AIT by differentiating between cross-reactivity and co-sensitization [16,43]. The use of CRD with purified natural or recombinant allergens has revealed that a significant number of polysensitized patients have IgE against highly cross-reactive panallergens (ranging from 10% for calcium binding proteins to around 40% for profilins) [44]. These observations define CRD as a complex but promising technology that will probably replace conventional ssIgE assays in the near future. Nevertheless, there is a need for prospective trials to assess the value of CRD for selecting (i) patients for AIT and (ii) the allergens to be administered [45].

Justicia *et al*. performed a study on sensitization to pollen from grasses and the olive tree (which have overlapping pollination periods in Spain: April to June and May to July, respectively) in 1263 patients with positive SPTs for both [46]. The 88 allergists involved in the study were first asked to define their choice of the AIT composition on the basis of the SPT results alone: grass pollen only in 18% of cases, both grass and olive pollen in 73% and olive pollen only in 9%. The patients’ serum samples were then assayed for sslgE against Ole e 1 and Phl p 1 and the allergists were invited to review the IgE titres and again recommend the most appropriate AIT. This resulted in the choice of grass pollen in 30% of patients, both grass and olive pollen in 35%, olive pollen only in 16% and no AIT in 19%. Importantly, the “before IgE” and “after IgE” recommendations differed for 55% of the patients. This results shows that knowledge of sensitization status has a major impact on treatment decisions.

A key remaining issue in the detection of sensitizations is when to rely on SPTs alone and when to investigate further with ssIgE assays and CRD. The latter technique may be of value in patients with positive SPTs for two or more related allergens. The indications for *in vitro* diagnostic tests are the same as for SPTs. *In vitro* diagnostic tests usually show higher sensitivity but lower specificity and therefore are performed whenever SPTs do not provide reliable results.

### Treatment approaches in polysensitized subjects and polyallergic subjects

In a recent review, Calderon *et al*. summarized the conventional treatment strategies for polysensitized subjects in North America and Europe [47]. In the USA, allergists tend to prescribe (subcutaneous) AIT formulations comprising allergens that correspond to all important allergies (or sometimes only sensitivities) identified in SPTs. As a result, the AIT formulations used in the USA contain an average of 8 components [48]. Calderon *et al*. stated that multi-allergen immunotherapy (whether administered subcutaneously or sublingually) in polysensitized patients requires more supporting evidence from well-designed, well-powered double-blind, placebo-controlled clinical trials to validate its efficacy in practice. In Europe, the polysensitized patient is typically treated with the single allergen (or at most two or three allergens) deemed to be most clinically relevant. A number of observational studies performed in Europe have assessed the management of polysensitized patients. For example, the fifth POLISMAIL study was an open assessment of the clinical efficacy of SLIT in 51 polysensitized children (mean age: 11.8) with AR and/or mild-to-moderate AA [49]. One, two and three allergens were prescribed in 82%, 8% and 6% of cases, respectively (with missing data in 4%). Sublingual AIT was associated with significant reductions in ocular, nasal, and bronchial symptom scores (p <0.01) and rescue medication use (p <0.01). There were no systemic reactions in the single-allergen group or the multi-allergen group. The authors concluded that polysensitization should not represent a counter-indication for prescribing AIT with one or two allergen extracts (preferably administered separately and at high dosage levels). In an overview of the six POLISMAIL studies, Ciprandi *et al*. identified five important aspects for added-value molecular diagnoses and optimal AIT in polysensitized patients: (i) rapid, comprehensive assessment of the sensitization profile, (ii) knowledge of symptoms or diagnostic results caused by cross-reactivity, (iii) interpretation of complex sensitization results (e.g. panallergens), (iv) more accurate identification of candidates for AIT and (v) potential cost reductions through optimization of AIT [33].

Of course, choosing the right allergen (s) for AIT is essential. Sastre *et al*. looked at whether a molecular diagnosis would change the allergen prescription in AIT in a group of 141 patients with pollen-induced AR [43]. The patients were tested with SPTs and the ISAC® microarray-based panel of allergens before and after the prescription of AIT. In 54% of cases, the molecular diagnosis changed the indication for AIT. The degree of agreement varied according to the allergen in question (being higher for olive and cypress extracts and lower for grass pollen extract). The authors considered that the poor levels of agreement were due to the prescription of AIT on the basis of SPTs with a substantial proportion of false-negative results. The degree of agreement was also worsened by the presence of sensitization to profilin and/or polcalcin.

## Conclusions

### Characterization of sensitizations

Polysensitization is highly prevalent in patients being treated for allergies, although the prevalence depends on the population and the region and changes over time. A supposedly monosensitized patient may merely have been screened against a small or inappropriate panel of allergens and thus the presence of a clinically relevant allergy to another allergen should still be considered if supported by clinical evidence. Hence, we suggest that there is a need for (i) further standardization of SPT allergen panels used in the same geographical or climatic area, (ii) an increase in the number of allergens used in SPT panels, (iii) the customization of the allergen panels for particular geographical or climatic areas and (iv) use of CRD in patients with positive SPTs for two or more related allergens.

### The prevalence of polysensitization

An overall assessment of the epidemiological and clinical trial data suggests that between 50% and 80% of patients consulting allergists are polysensitized. The exact prevalence depends on the population and the region. Patients tend to gain sensitizations over time.

### The relationships between polysensitization, polyallergy and treatment efficacy

The definitions of monosensitization, paucisensitization and polysensitization are of clinical relevance. Polysensitized patients do not necessarily have polyallergy, whereas all polyallergic patients will be polysensitized. Polysensitization develops over time and may be associated with disease severity. The current evidence suggests that single-allergen AIT is effective in polysensitized (but not necessarily polyallergic) patients. The role of CRD for guiding treatment should also be investigated. A detailed molecular diagnosis will add value when determining whether AIT is appropriate for a given patient and, if so, which allergen (s) should be administered. However, there is clearly a need for well-powered, specifically designed clinical studies comparing the efficacy and safety of (i) single-allergen AIT, (ii) AIT with two or three allergen extracts (preferably delivered separately and at high dosage levels) and (iii) AIT with a high number of allergen extracts (e.g. 8 to 10). Overall, there is a need to develop algorithms or decision trees for the choice of the allergen (s) to be included in AIT for polyallergic patients.

## Competing interests

Michel Migueres has received consulting fees, honoraria for lectures and/or research funding from Stallergenes, Novartis, Astra Zeneca, Chiesi, Pierre Fabre Santé Ignacio Dávila has received consulting fees, honoraria for lectures and/or research funding from Stallergenes. Franco Frati is an employee of Stallergenes Italia SRL. Angel Azpeitia is an employee of Stallergenes Iberica SA. Yasmine Jeanpetit and Michèle Lhéritier-Barrand are employees of Stallergenes SA. Cristoforo Incorvaia has received consulting fees from Stallergenes Italia. Giorgio Ciprandi has received consulting fees from Stallergenes Italia. This work was supported by an educational grant from Stallergenes.

## Authors’ contributions

MM, IDG, FF, AA, YJ, MLB, CI and GC all made substantial contributions to the identification and review of relevant literature, drafting the manuscript and/or revising it critically for important intellectual content. All the authors have given final approval of the version to be published.
